# Regulatory effect of bacterial melanin on the isoforms of new superoxide-producing associates from rat tissues in rotenone-induced Parkinson’s disease

**DOI:** 10.1186/s12868-023-00838-9

**Published:** 2023-12-20

**Authors:** Margarita Danielyan, Kristina Nebogova, Ruzan Simonyan, Anichka Hovsepyan, Zubeida Avetisyan, Karen Simonyan, Gegham Simonyan, Vahagn Khachatryan, Kristine Karapetyan

**Affiliations:** 1 Orbeli Institute of Physiology NAS RA, Yerevan, Armenia; 2 Institute of Biochemistry NAS RA, Yerevan, Armenia; 3 Scientific and Production Center “Armbiotechnology” NAS RA, Yerevan, Armenia

**Keywords:** Brain, Small intestine, Parkinson’s disease, Bacterial melanin, Superoxides, Associates

## Abstract

According to recent research, selective neuronal vulnerability in Parkinson’s disease (PD) results from several phenotypic traits, including calcium-dependent, feed-forward control of mitochondrial respiration leading to elevated reactive oxygen species and cytosolic calcium concentration, an extensive axonal arbor, and a reactive neurotransmitter. Therefore, antioxidant therapy is a promising direction in the treatment of PD. In vitro studies have indicated the survival-promoting activity of bacterial melanin (BM) on midbrain dopaminergic neuron cultures. It has been established that BM has a number of protective and anti-inflammatory properties, so there is a high probability of a protective effect of BM in the early stages of PD. In this study, PD was induced through the unilateral intracerebral administration of rotenone followed by bacterial melanin. Tissues (brain, lungs, and small intestine) from the observed groups underwent isolation and purification to extract isoforms of new thermostable superoxide (О_2_^−^)-producing associates between NADPH-containing lipoprotein (NLP) and NADPH oxidase-Nox (NLP-Nox). The optical absorption spectral characteristics, specific amounts, stationary concentration of the produced О_2_^−^, and the content of NADPH in the observed associates were determined. The optical absorption spectra of the NLP-Nox isoforms in the visible and UV regions in the experimental groups did not differ from those of the control group. However, compared with the control group, the specific content of the total fractions of NLP-Nox isoforms associated with PD groups was higher, especially in the small intestine. These findings suggest that the described changes may represent a novel mechanism for rotenone-induced PD. Furthermore, bacterial melanin demonstrated antioxidant properties and regulated membrane formation in the brain, lung, and small intestine. This regulation occurred by inhibiting the release of new membrane-bound formations (NLP-Nox associates) from these membranes while simultaneously regulating the steady-state concentration of the formed О_2_^−^.

## Introduction

While dopaminergic neuronal loss is a histopathological finding in Parkinson’s disease (PD), the disease is multifactorial and has several determinants. According to a review article by Pang et al. [1], selective neuronal vulnerability in PD results from several phenotypic traits, including calcium-dependent, feed-forward control of mitochondrial respiration leading to elevated reactive oxygen species and cytosolic calcium concentration, an extensive axonal arbor, and a reactive neurotransmitter. These traits increase vulnerability to genetic mutations associated with PD, age, and environmental toxins. In recent years, there has been increased attention on the role of antioxidants in regulating free-radical processes and treating various diseases. Free radicals form in the body as a byproduct of oxygen metabolism in tissues. The brain, consuming about 20% of the body’s oxygen, undergoes intense oxidative processes and is characterized by a low level of antioxidant protection [2]. In PD, there is a decrease in the activity of the antioxidant system, disrupting the balance between pro-oxidant and antioxidant processes [3]. PD is a slowly developing pathology of the central nervous system, exhibiting both motor and non-motor symptoms. Research indicates that the primary cause of PD is the progressive destruction and death of neurons producing the neurotransmitter dopamine, located in the substantia nigra pars compacta (SNc), along with their nerve endings in the striatum [4]. SNc neurons play a crucial role in motor learning and related behavior [5].

The relationship between peripheral organs and the brain in PD is an area of active research. PD is now understood to be a multi-system disorder that affects not only the central nervous system but also peripheral organs. There is evidence suggesting that PD affects a chain of neurons in autonomic pathways, leading to dysfunction in various peripheral organs, such as the cardiovascular, skin/sweat gland, urinary, gastrointestinal, pupillary, and neuroendocrine systems [6]. Additionally, studies have suggested a potential link between PD and peripheral neuropathy, a common neurologic condition [7]. Furthermore, inflammation and immune dysfunction have been implicated in the development of various non-motor symptoms in PD, such as sleep and gastrointestinal dysfunction, highlighting the relevance of the gut-brain axis in the disease [8, 9].

Inflammatory factors are shown to contribute to the death of dopaminergic neurons in the substantia nigra, and the acceleration of their death is triggered by processes involving oxidative stress [10]. The substantia nigra is particularly prone to generating free radicals, as hydrogen peroxide forms during the metabolism of dopamine and its autooxidation. One reason for neuron death, resulting from the activation of lipid peroxidation, may be an increase in membrane permeability to ions. In PD, the substantia nigra is characterized by an elevated iron ion content, serving as a catalyst for oxidative processes, and a low glutathione content, which possesses antioxidant properties [11]. The mechanisms responsible for the degeneration of nigrostriatal dopaminergic neurons remain unknown. However, oxidative stress, arising from an imbalance in the oxidant and antioxidant status, is believed to play a significant role in dopaminergic neurotoxicity. Increased immunoreactivity for the NADPH oxidase catalytic subunits Nox1, Nox2, and Nox4 has been reported in the brains of PD patients. Nevertheless, the connections between NADPH oxidases and biological processes contributing to neuronal death are not well understood [12].

On the other hand, new superoxide-producing thermostable associates from animal cell membranes, specifically intracellular formation membranes between NADPH-containing lipoprotein (NLP) and NADPH oxidase (Nox), known as NLP-Nox, have recently been prepared [13]. NADPH-containing lipoprotein (NLP) serves as a substrate for these enzymes within the composition of these associates. Under aerobic conditions, the production of О_2_^−^ by the isoforms of these associates occurs continuously, utilizing electrons from NLP to generate O_2_.

One of the promising directions in the treatment of PD is antioxidant therapy [14], especially at the initial stage of the disease. Additionally, various types of neuroprotectors have been successfully employed in the treatment of neurodegenerative diseases in recent decades, leading to the accelerated restoration of lost functions in the central nervous system (CNS) structures. In the current study, bacterial melanin (BM) was utilized for this purpose. BM was obtained on the basis of the production strain of *Bacillus thuringiensis* under the action of nitrosoguanidine. The water-soluble BM obtained through biotechnological means holds potential as a biological medical product for the treatment of neurodegenerative diseases, including PD [15]. The molecular weight of the purified melanin determined by SDS-PAGE was 4 kDa and the electromagnetic spin resonance spectrum of the purified microbial melanin was a slightly asymmetric singlet without hyperfine structure with about 7 Gauss width of the line between points of the maximum incline and g = 2.006. The concentration of paramagnetic centers in melanin is 0.21 × 10(18) spin/g [16].

Literature data indicate that BM has been utilized in various physiological studies to facilitate the rapid recovery of impaired motor functions in rats following the destruction of certain CNS structures involved in the organization of the animal’s motor behavior. It has been determined that BM exhibits high biological activity and a biostimulating effect, and at low concentrations (4.5–6 mg/ml), it does not induce microgliosis or have toxic side effects [17, 18]. BM may promote the survival of neurons in the substantia nigra in the presence of toxic factors. In vitro studies have indicated the survival-promoting activity of bacterial melanin on midbrain dopaminergic neuron cultures [19]. BM has the potential to enhance the survival of neurons in SNc under the influence of toxic factors, suppress inflammatory processes, and stimulate regeneration processes in nervous tissue [15, 20]. It has been established that BM possesses protective and anti-inflammatory properties [21], suggesting a high probability of BM exerting a protective effect in the early stages of PD as an antioxidant agent.

In the literature available to us, we have not encountered experimental studies devoted to the study of the antioxidant properties of BM. Studies reporting the potential use of BM are mostly in the developmental stage.

Melanins from various sources exhibit significant antioxidant activity [22, 23]. The role of melanin as a scavenging or quenching molecule on superoxide anions, and singlet oxygen species has been discussed by Tada M et al. [24] who used ESR and spectrophotometric methods to show that melanin potently interacts with reactive oxygen species that are generated in certain physiological reactions. The high content of paramagnetic centers allows melanins to deactivate radicals due to the large electron absorption capacity of these compounds [25]. The presence of paramagnetic centers in BM may be a prerequisite for the antioxidant effect of this pigment.

The aim of this study is to determine the quantitative and qualitative changes in the total fractions of the О_2_^−^-producing associates in rat tissues (brain, lungs, and small intestine) in a rotenone-induced PD and the influence of BM as a potential regulatory agent.

## Materials and methods

The rotenone model was employed as an experimental model of PD, recognized for its reliability in studying mechanisms of neuronal damage, particularly up to 4 weeks of survival [26]. Rats were categorized into three groups, each consisting of 5 rats: (1) the control group (C group), (2) the PD model induced by unilateral intracerebral administration of rotenone and maintained for 4 weeks (PD group), and (3) PD rats administered bacterial melanin (PD + BM group). Rotenone administration was conducted under anesthesia (pentobarbital, 40 mg/kg, i.p.) at a dose of 12 µg rotenone in 0.5 µl DMSO, delivered at a rate of 1 µl/min into the medial forebrain bundle, following stereotaxic atlas coordinates (AP + 0.2; L ± 1.8; DV + 8 mm) [27]. The BM solution was administered to the experimental animals the day after the rotenone injection at a concentration of 6 mg/ml (i.p.). The volume of the injected solution was calculated based on the optimally tolerated dose (0.17 g per kilogram of body weight) at the time of introducing the BM solution. Before the experiments, white rats (230 ± 30) underwent a week of acclimation to laboratory conditions while being fed standard rodent food. The acclimated rats were fasted for 12 h with free access to water before receiving the rotenone injection. The animals were kept under consistent conditions throughout the entire 4-week postoperative period preceding the acute experiment. After 4 weeks, the animals were fasted, anesthetized with pentobarbital (40 mg/kg, i.p.), and dissected. The experimental protocol corresponded to the conditions of the European Communities Council Directive (2010/63/ UE) and was approved by the Ethics Committee of Yerevan State Medical University after Mkhitar Heratsi (IRB Approval N4, November 15, 2018).

### Isolation and purification of a total fraction (О_2_^−^-producing associates NLP-Nox) from cell membranes and membranes of intracellular formations of the brain, lung, and small intestine of the rats in the abovementioned groups

The isoforms of О_2_^−^-producing associates, NLP-Nox, from the rat’s brain, lung, and small intestine were isolated and purified using a universal method [13]. The process involved freezing and defreezing the aqueous homogenate of these organs (to 10 g), followed by incubation at pH 9.5 and 37 °C for 1.5 h with 50 µM ferrihemoglobin as a stimulator for releasing NLP-Nox from membranes to the solution. After centrifugation of this mixture at 5000 × g for 10 min, the pH of the supernatant was adjusted to 4.8. The precipitate of the NLP-Nox fraction was soluble in water (forming a weak opalescent solution at pH 9.5). After centrifugation, the supernatant underwent ion-exchange chromatography on cellulose DE-52 at pH 9.5. The Nox-NLP associates were not absorbed on this column and were eluted freely. Following the concentration of these total fractions of NLP-Nox, gel filtration on a separate column of Sephadex G-200 at pH 9.5 was performed. The NLP-Nox fractions were eluted with a symmetrical elution diagram, and after deionization, the isoforms of the NLP-Nox associate were incubated in boiling water for 10 min. After centrifugation, the supernatant was subjected to vacuum lyophilization. The isoforms of the prepared NLP-NOX associate were weighed and stored under anaerobic conditions at -10 °C. Electrophoresis of the isoforms of Nox-NLP associates was conducted on a 7% or 10% Polyacrylamide Gel (PAGE) for proteins with acidic or basic characteristics.

### Determination of NADPH in the composition of isoforms of the total fraction of NLP-Nox from the C, PD and PD + BM groups

The presence of NADPH in the total fraction of the isoforms of NLP-Nox was assessed using a spectrofluorimetric method. This involved determining the fluorescence intensity (F) in relative units at 430 nm with excitation at 370 nm [28].

### Determination of a lipid component in the composition of total fraction of NLP-Nox

The lipid composition of the above-mentioned isoforms of NLP-Nox was determined by measuring the lipid peroxidation product malondialdehyde (MDA) [29].

### Determination of the stationary concentration of О_2_^−^, produced by NLP-Nox isoforms presented above

The stationary concentration of О_2_^−^ produced by the total fraction of isoforms of the NLP-Nox associate from the aforementioned rat tissues was determined using the adrenaline method. The maximal optical absorbance of adrenochrome (at 500 nm) is formed during the oxidation of adrenaline by the produced О_2_^−^ [30]. Simultaneously, the stationary concentration (M) of produced О_2_^−^ is equivalent to the concentration of formed adrenochrome, with the molar extinction (E) up to 750 M-1 cm-1. The value of A500/E, representing the stationary concentration (M) of О_2_^−^ produced by these NLP-Nox associates, was determined in the homogeneous phase (in solution) and in the gas phase (during the blow of molecular oxygen, 0.1 atm of water solutions of NLP-Nox). The optical absorbance of adrenochrome formed during the oxidation of adrenaline by atmospheric oxygen served as a control. The specific content (mg/g tissue) of NLP-Nox was determined by weighing after deionization and vacuum lyophilization. Throughout the investigation, cellulose DE-52 (Whatman, England), Sephadex G-200 (Pharmacia, Sweden), adrenaline (Sigma, USA), the spectrophotometer Cary 60 (USA), spectrofluorimeter Perkin-Elmer (USA), and centrifuges K-70D and K-24 Janetzki (Germany) were utilized.

The statistical treatment of the received results was carried out using the variation statistical method of Student-Fisher, by determining the criteria of reliability “p”, m ± M, n = 6.

## Results

**Fig. 1:**
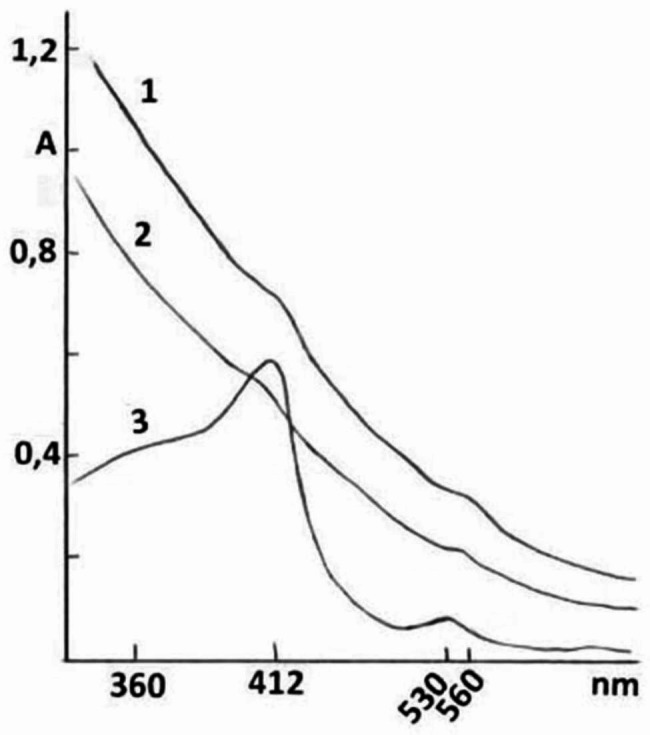
The optical absorption spectra of the weak opalescent aqueous solutions (at pH 9,5) of total fraction of isoforms of NLP-Nox from brain (1), small intestine (2) and lung (3) tissues. The forms of optical absorption spectra of the isoforms of NLP-Nox from C, PD and PD + BM groups (n = 5in each group) do not differ

The isoforms of the total fraction of О_2_^−^-producing associates, NLP-Nox, from the rat’s brain, lung, and small intestine tissues in the C, PD, and PD + BM groups did not undergo PAGE electrophoresis; they remained in an aggregated state at the entry of the gel tubes. The purity of these associates is indirectly supported by the absence of strips of acidic and basic water-soluble proteins during electrophoresis of the opalescent solutions of these associates in 7% or 10% PAGE tubes. Furthermore, the symmetry of the elution diagrams of the total fractions of the isoforms of the Nox-NLP associate after gel-filtration through Sephadex G-200 and the unchanged optical spectral index (A280/A400) also indicates the purity of these associates. The presented isoforms from the C, PD, and PD + BM groups practically retain their nativity and О_2_^−^-producing activity even after being subjected to boiling water for 10 min for denaturation of traces of other potential proteins. The optical absorption spectra of the total fractions of the isoforms of NLP-Nox from the brain, lung, and small intestine membrane formations in the C, PD, and PD + BM groups in both oxidized and reduced states at pH 9.5 exhibit characteristic absorbance at 412 nm, 530 and 560 nm (Fig. 1).

The lipid component content, specifically malondialdehyde, in the presented NLP-Nox associates from PD rat groups is higher by 20–22% compared to that in control rats. In the oxidized state, characteristic maxima in the optical absorption spectra of the isolated NLP-Nox were observed at 412, 530, and 560 nm. In the reduced state (by potassium dithionite), maximal optical absorbance was observed at 418, 540, and 558 nm. These forms of optical absorption spectra of NLP-Nox associates are indicative of the optical absorption spectra of Nox in the composition of isoforms of these associates’ solutions of NLP-Nox. The shapes of these spectra do not differ for NLP-Nox from the C, PD, and PD + BM groups, and therefore they are not presented. In the UV regions, the maximal optical absorbance of the presented associates at 260 nm, 275 nm, and 280 nm (characteristic absorptions for proteins) and the forms of the optical absorption spectra of isoforms of the total fraction of NLP-Noxrats’ tissues in PD groups are similar to the spectra of C and PD + BM groups and are not presented.

The above mentioned isoforms of NLP-Nox associates are enzymatic biological systems in which the substrate is not free NADPH but the NADPH in the composition of NLP. The nicotinamide moiety of NADPH absorbs light of wavelength 340 **±** 30 and emits fluorescence at 460 **±** 50 nm [31]. These associates are localized slightly deeper than the surface of biomembranes and act as “biological microguns”, which shoot electrons, for the one-electron reduction of molecular oxygen to О_2_^−^, specifically in aerobic conditions. The isoforms of NLP-Nox associates (enzymes) are thermostable and in aerobic conditions continuously produce О_2_^−^. The mechanism of О_2_^−^ production by these associates is conditioned by the transfer of electrons from NADPH in the composition of NLP-Nox to Fe(III) of the heme group of this Nox, and then to O_2_, reducing it to О_2_^−^. On the other hand, when blowing water solutions of the NLP-Nox associate, a gas-phase О_2_^−^ is formed and can be transferred with silicone or glass tubes. Essentially, oxygen stabilizes the gas-phase О_2_^−^ through the formation of a coordination bond between O_2_ and О_2_^−^. It is known that gas-phase О_2_^−^ forms in the air through various processes, including the reduction of molecular oxygen with trace negative metal ions, electrochemical methods using a ‘gas-phase superoxide generator,’ and also due to the influence of Earth’s crust radioactivity [32, 33]. The gas-phase О_2_^−^ generated from biosources (associates of NLP-Nox localized on biomembranes) can find applications in various fields of biochemistry and biomedicine as a monocomponent, pure О_2_^−^. The specific contents of NLP-Nox in tissues are shown in Table 1.

**Table 1:** Specific contents of the isoforms of NLP-Nox (mg) in 1 g tissues (mg/g) in the C, PD, and PD + BM groups

Tissue	C	PD	PD + BM
Brain	82,6 ± 4,3	90,7 ± 5,4(p < 0,001)	84.1 ± 3,3(p < 0,005)
Small intestine	21,9 ± 1,1	81,6 ± 4,5 (p < 0,001)	30,1 ± 3,1(p < 0,001)
Lung	10,32 ± 0,3	14,2 ± 0,05 (p < 0,005)	11,4 ± 0,3 (p < 0,001)

The observed increase in the amount of these associates from tissues in the PD groups may be attributed to the heightened lipid peroxidation of the membranes [34, 35] and the deterioration of the binding band for the association with the membranes. Consequently, the release of NLP-Nox from the membranes to the solution was observed in the PD groups. This phenomenon could potentially serve as a novel diagnostic test for PD. The stationary concentration of О_2_^−^ produced by the isoforms of the total fraction of the NLP-Nox associates at 20 °C is detailed in Table 2.

**Table 2:** The stationary concentration (x 10^− 4^ M) of produced О_2_^−^ by 1 mg total fraction of the isoform of NLP-Nox associates from rat tissues at 20 °C (M/mg) in the C, PD and PD + BM groups

Tissue	C	PD	PD + BM
Brain	4,3 ± 0,2	3,8 ± 1,1 (p < 0,01)	5,1 ± 0,2 (p < 0,001)
Small intestine	83,2 ± 4,3	70,5 ± 2,4 (p < 0,005)	78,6 ± 2,2(p < 0,005)
Lung	21,8 ± 2,1	13,5 ± 0,4 (p < 0,001)	17,7 ± 1,6 (p < 0,001)

## Discussion

Recent research suggests that selective neuronal vulnerability in PD results from several phenotypic traits, including calcium-dependent, feed-forward control of mitochondrial respiration leading to elevated reactive oxygen species and cytosolic calcium concentration, an extensive axonal arbor, and a reactive neurotransmitter. These traits increase vulnerability to genetic mutations associated with PD, age, and environmental toxins [36]. Oxidative stress and hyperglycemia in metabolic diseases lead to adverse neurophysiological phenomena, including neuronal loss, synaptic dysfunction, and improper insulin signaling, resulting in PD [37]. Therefore, while dopaminergic neuronal loss is a significant factor in PD, it is not the only cause, and the disease is multifactorial [38].

The oxidative pathway of dopamine metabolism in the human brain results in the formation and accumulation of neuromelanins in the cytoplasm of most nigrostriatal dopaminergic neurons. Neuromelanins exhibit natural antioxidant properties, effectively suppressing lipid peroxidation [39]. When nigrostriatal dopaminergic neurons are damaged, neuromelanins are released from cells. Free extracellular neuromelanin induces microgliosis, identified as a primary cause of PD [40]. In the absence of microglia, extracellular neuromelanin is non-toxic to neurons. However, the release of neuromelanin from compromised neurons triggers microglial activation and the neurodegenerative process. Studies using BM have demonstrated that this substance does not induce microgliosis and has no toxic side effects [17]. In our study, rotenone was employed to create an experimental animal model of PD that simulates and induces symptoms akin to Parkinson’s disease, including motor and cognitive impairment [26]. Rotenone, a pesticide, inhibits mitochondrial complex I activity, creating an oxidative stress environment in the cell. The involvement of rotenone in induced oxidative damage supports the exploration of antioxidant therapy in Parkinson’s disease [41, 42]. In the SNc of the brain, one of the by-products of enzymatic reactions is hydrogen peroxide, which further breaks down into highly toxic hydroxyl radicals. Typically, these radicals are neutralized by the glutathione system. However, the activity of antioxidant systems decreases with age, contributing to the age-related decline in the resistance of dopaminergic nigrostriatal neurons to pro-Parkinsonian factors and the progressive increase in PD patients. The concept of oxidative stress in the pathochemical mechanisms of neuronal damage in PD defines an avenue for pathogenetic therapy with antioxidants [43]. The high content of paramagnetic centers in melanins enables them to deactivate radicals due to their high electron absorption capacity. Melanin has been demonstrated to play a role in DNA repair, act as a modulator in crucial cellular metabolism systems such as photo- and radioprotection, neutralize products of lipid peroxidation, and participate in neurotransmitter processes during various pathological disorders affecting the functional structures of neurons [25].

According to our data, the fluorescence intensity in relative units (F) of the NADPH in the compositions of total fractions of the NLP-Nox associates from tissues of the presented groups (C, PD, PD + BM) was directly proportional to the stationary concentration of produced О_2_^−^in the PD group. It is plausible that, as a consequence of lipid peroxidation of lipoproteins in the PD group in vivo, the decrease in the amount of NADPH in the composition of NLP results in a reduction in the stationary concentration of produced О_2_^−^.

The monocomponent gas phase О_2_^−^ generated enzymatically from the NLP-Nox associates mentioned above, which regulates the stationary concentration of О_2_^−^, can be utilized via an oxygen mask for the treatment of lung infection diseases in experimental animals and potentially in clinics in the future. The preferential aspects of this approach lie in the monocomponent nature and regulated stationary concentration of enzymatically produced О_2_^−^. In these associates, the substrate used was NLP within their composition. Importantly, no denaturation or decrease in superoxide production was observed after incubating the aqueous solution of these associates in boiling water for 10–12 min. The higher thermostability of the О_2_^−^-producing NLP-Nox from these associates may be attributed to brief, nanosecond pulses of temperature increase up to 280-300oC, facilitating the transmission of redox metabolic processes [44]. The fundamental significance of the obtained results includes: (1) the immediate mechanism of О_2_^−^ production by the NLP-Nox associates on biomembranes, (2) the formation of a monocomponent and regulating the stationary concentration of the gas phase О_2_^−^from solutions of these NLP-Nox, and (3) the localization of NLP-Nox isoforms in the deeper layers of biomembranes, utilizing NLP-bound NADPH as the substrate for an uninterrupted supply of superoxide radicals, supporting continuous aerobic metabolic processes. These NLP-Nox isoforms function as uninterrupted biological micro-automates, facilitating the transfer of electrons to molecular oxygen for its one-electron reduction, resulting in О_2_^−^. The production of О_2_^−^ stops only under anaerobic conditions. The applied significance of the obtained results encompasses: (1) qualitative and quantitative changes in these associates, serving as new sensitive diagnostic tests for various pathological states and diseases, including gastrointestinal diseases, neurodegenerative diseases, and PD; and (2) the utilization of gas-phase О_2_^−^, which can be employed as an antimicrobial and anticancer agent, as well as a stimulator of bone marrow stem cell proliferation, etc.

Analysis of the data led to the conclusion that BM exhibits antioxidant effects and reduces the formation of lipid peroxides. The BM used in this study is proposed as a potential therapeutic agent for preventing PD and/or its progression [45]. In summary, the total fractions of isoforms of the NLP-Nox associates from the C, PD, and PD + BM groups represent a new О_2_^−^-producing, thermostable associate from membrane formations in the rat’s brain, lung, and small intestine. These associates produce О_2_^−^ through an immediate mechanism, displaying corresponding quantitative and qualitative changes in C, PD, and PD + BM rats, thus serving as a new diagnostic test for PD. BM emerges as an effective preparation for regulating the indices presented above in PD.

In conclusion, there is evidence suggesting that PD impacts not only the central nervous system but also peripheral organs. Nevertheless, the relationship between peripheral organs and PD remains an area of ongoing research, requiring more studies to fully comprehend the complex interplay between peripheral organs and the brain in PD.

## Data Availability

All data generated and analyzed during this study are included in this article. The datasets used and/or analyzed during the current study are available from the corresponding author on reasonable request.
